# Utility of ultrasound guided versus conventional fine needle aspiration cytology in diagnosing breast malignancies among patients with palpable breast lumps at Bugando Medical Centre, Mwanza Tanzania

**DOI:** 10.11604/pamj.2021.39.133.22663

**Published:** 2021-06-16

**Authors:** Tresphory Bonephace Kamushaga, Geofrey Charles Giiti, Benson Richard Kidenya, Patrick Sitati Ngoya, Peter Fabian Rambau

**Affiliations:** 1 Department of Surgery, Catholic University of Health and Allied Sciences, Mwanza, Tanzania,; 2 Department of Biochemistry, Catholic University of Health and Allied Sciences, Mwanza, Tanzania,; 3 Department of Radiology, Catholic University of Health and Allied Sciences, Mwanza, Tanzania,; 4 Department of Pathology, Catholic University of Health and Allied Sciences, Mwanza, Tanzania

**Keywords:** Conventional, ultrasound guided, fine needle aspiration cytology, breast lump

## Abstract

**Introduction:**

breast lump is the commonest presentation for both benign and maligant breast conditions. Both ultrasound guided and conventional fine needle aspiration cytology (FNAC) have been used for diagnosing of breast malignancy among patients with palpable breast lumps. This study compared diagnostic utility of ultrasound guided versus conventional FNAC in diagnosing breast malignancies among patients with palpable breast lumps at Bugando Medical Centre.

**Methods:**

this was a hospital based cross sectional study with a follow up component that combined both retrospective data (from January 2017 to June 2018) and prospective data (from July 2018 to June 2019).

**Results:**

during the study, total of 354 patients (male; female = 1: 32) were enrolled in the study. A total of 134 (37.9%) patients had malignant lesions while 220 (62.1%) of patients had benign lesions confirmed on histology. The diagnostic utility (sensitivity, specificity, positive predictive value, negative predictive value and diagnostic accuracy) for conventional FNAC was 86.7%, 95.7%, 93.5%, 91.1% and 92.0% with an 8% error margin versus ultrasound guided FNAC all were 100% with a 0% error margin respectively.

**Conclusion:**

both ultrasound guided and conventional FNAC show almost perfect agreement with histology. However, ultrasound guided FNAC has a higher diagnostic utility relative to conventional FNAC in diagnosing breast malignancies.

## Introduction

Breast cancer is the most common malignant neoplasm and the second cause of death from cancer in women, with more than one million cases occurring worldwide annually [1,2]. A breast lump is the most common clinical presentation for both benign and malignant breast lesions [3]. With growing awareness in the general population, especially about breast cancer, a lump in the breast causes physical, emotional and psychological trauma to the patient and family members [4]. Therefore, a distinction of benign from malignant breast lump is of paramount importance for proper management [1,4]. Appropriate diagnosis of breast lesions not only saves the patient from unnecessary physical, emotional and psychological trauma, but also relieves the health services from undue burden. On the other hand, a definitive pre-operative diagnosis of malignant lesion provides opportunities for patient´s counseling and planning of possible single-stage surgical treatment [1,4]. Every patient in cancer prone age with palpable breast lump must be thoroughly evaluated and investigated. At present, a wide range of diagnostic imaging modalities are available for the evaluation of breast lumps. These include ultrasound scanning, mammography and recently magnetic resonance imaging (MRI) and contrast enhanced ultrasound [5]. However, in a poor resource setting, these are limited by their availability and cost, and therefore not all patients can benefit from them. Conventional open biopsy is considered as the gold standard for confirming the diagnosis, however, it has significant morbidity, it is costly, invasive, time consuming and exposes the patient to unnecessary anxiety and more than one surgical procedure [6]. It also has high chance for spread of malignancy and it is lengthy, and the time taken to get histology results can lead to delay in definitive diagnosis and treatment [3,7].

There is an important need of developing a method for establishing the diagnosis pre-operatively, which is cost effective, least invasive and not disturbing to the patient with accuracy comparable to that of open biopsy [3]. Triple assessment (clinical examination, breast imaging and FNAC) is a recommended diagnostic modality is not practicable in many Tanzanian hospitals because of lack of mammography screening services. Fine needle aspiration cytology of breast lumps either ultrasound guided or conventional is an important part of triple assessment of palpable breast lumps [8-16]. It bridges the gap between clinical evaluation and final surgical pathological diagnosis in majority of cases and helps to reduce unwanted surgeries [8-12,14-16]. Fine needle aspiration cytology has been routinely practiced in the developed world as an alternative to conventional open biopsy in the pre-operative diagnosis of breast lumps [8-16]. It is simple, reliable, reproducible, less traumatic and cost effective in terms of money and time and its diagnostic accuracy has been reported to exceed 99% [12-14,16]. Fine needle aspiration cytology has been practiced in some centers in Tanzania where the facilities and experts are available [17]. This study was conducted to compare diagnostic utility of ultrasound guided FNAC and that of conventional FNAC in the diagnosis of breast malignancies among patients with palpable mass at Bugando Medical Centre.

## Methods

**Study design:** this was a hospital based cross-sectional study with a follow up component, and combined both retrospective and prospective methods. Retrospective data obtained data from January 2017 to June 2018 (over 18 months) and prospective data from July 2018 to June 2019 (over 11 months). The study was conducted in the general surgical outpatient´s clinics and wards, radiology and oncology, histopathology laboratory and medical records departments of Bugando Medical Centre (BMC). BMC is the only tertiary institution serving the Northwestern Zone of Tanzania, serving a population of about 16 million people. It has a 890 bed capacity, located in Mwanza, Tanzania along the shores of Lake Victoria. It is also a teaching hospital for the Catholic University of Health and Allied Sciences (CUHAS).

**Study population:** study included patients with palpable breast lumps regardless of their age and sex who had conventional and ultrasound guided FNAC for palpable breast lumps, and subsequently had open biopsy or surgical management and histology results were available from January 2017 to June 2019. Patients with incomplete data were excluded from the study.The minimum sample size of this study was calculated using Buderer´s formula [18], where 354 patients were enrolled into the study. Convenient sampling was employed to select patients who met the inclusion criteria until the total number of patients needed for the study was reached. The study variables included, demographic data (age, sex, occupation, and education level), parity, and family history of breast cancer, marital status, and menopausal status, duration of illness, anatomical side and quadrant, size of breast mass, FNAC and histopathological results.

**Data collection:** data on each patient was collected and entered into a pretested coded questionnaire prepared of the study. Data entered in the questionnaire included demographic data (age, sex, occupation, and education level), parity, family history of breast cancer, marital status, and menopausal status, duration of illness, anatomical side and quadrant, size of breast mass, FNAC and histopathological results. For retrospective component, some of data was missing, for example size of breast mass was not well documented, and this component was not included in analysis, but for patients studied prospectively this information was included.

**Study sampling:** for the prospective part, all patients with palpable breast lumps were first evaluated clinically, followed by counseling of patients on both methods; ultrasound guided and conventional FNAC. Retrospectively, data was collected from operation theatre register book from January 2017 to June 2018 and selection was done conveniently based on inclusion criteria till sample size required for the study was achieved. Ultrasound guided and conventional fine needle aspiration cytology were aseptically done for the mass lesions using 23G needle and 5ml syringe by aspiration technique under local anaesthesia. Two slides of thin films were prepared from aspirates by principal investigator, one fixed in 95% ethanol for PAP stain, and one air dried for May Grunwald Giemsa (MGG) stain. In case of cystic lesions, fluid was aspirated first followed by re-aspiration from the solid areas and the smears were evaluated in the routine way and the final diagnosis of the FNAC was reported. Findings of FNAC were correlated with data from open biopsy histology records. Fine needle aspiration cytology (FNAC) results were categorized into two groups as either benign or malignant. The tissue was fixed in 10% formalin, processed and paraffin embedded. The slides were stained using Haematoxylin and Eosin (H&E). Histopathological reports of biopsies taken from specimens removed after incisional, excisional biopsy or definitive surgery were also recorded as benign or malignant. Depending on the result of FNAC, all enrolled patients were scheduled for surgical intervention to obtain histopathological specimens, this was either incisional, excisional biopsy or definitive surgery like mastectomy and breast conserving surgery. The surgeries were performed either by a consultant surgeon or by principal investigator under the direct supervision of a consultant surgeon. Those who were confirmed to have breast malignancy were referred to Oncology Department for appropriate management. The management offered at Oncology department was chemotherapy, radiotherapy and/or hormonal therapy. Fine needle aspiration cytology (FNAC) findings of all the patients were recorded and compared with the open biopsy or histopathological results, hence, FNAC was tested as a diagnostic tool whereas open biopsy histological results was the “gold standard”.

**Statistical data analysis:** data analysis was performed using STATA version 13, the median (including the Interquartile Range) was calculated for continuous variables whereas proportions and frequency tables were used to summarize categorical variables. Fine needle aspiration cytology (FNAC) findings were tabulated against histopathological results as the gold standard. The sensitivity, specificity, positive predictive value, negative predictive value, accuracy error and kappa statistic were calculated for diagnostic agreement between each individual FNAC test compared to histology results.

**Ethical issues:** the approval to conduct this study was sought from the Joint CUHAS/BMC Research, Ethics and Review Committee before the commencement of the study (Approval No. CREC/293/2018). Permission to conduct the study was also obtained from the BMC Hospital Administration. Informed consent was obtained from each patient for the prospective study. Patients were informed that the information collected was maintained under strict confidentiality. For the retrospective part, institution waived the consent on condition the confidentiality of the patients was maintained. Patients who refused to consent or withdrew from the study were not jeopardized from accessing the medical care.

## Results

**Demographic characteristics:** this study enrolled 354 patients who presented with breast lumps, and among them 200 (56.5%) patients were studied retrospectively and the remaining 154 (42.5%) were studied prospectively. Three hundred and forty three (96.9%) were females and 11 (3.1%) were males with a male to female ratio of 1: 32. A total number of 134 (37.9%) patients had malignant lesions, whereas 220 (62.1%) of patients had benign lesions. The age of patients enrolled in this study ranged from 8 to 87 years with a median age of 40 years, Interquartile range (IQR) of 25 to 52 years. The median age of patients with malignant lesion was 51 years (IQR, 41 to 60 years) and that of patients with benign lesions was 29.5 years (IQR, 22 to 50 years). This difference was statistically significant (p<0.001). The peak age group of patients was 40-59 years constituted 39.0% of the cases.

**Conventional and ultrasound guided FNAC versus histological findings:** on conventional FNAC evaluation, a total number of 77 patients were diagnosed to have malignant lesions, while 123 patients had benign lesions, after histology the false positives and false negatives were 4.3% and 13.3% respectively (p<0.0001). From ultrasound guided FNAC, a total number of 51 and 103 patients were histologically confirmed malignant and benign respectively, there were no false positives or false negatives (p<0.0001) (Table 1). The diagnostic utility (sensitivity, specificity, positive predictive value (PPV), negative predictive value (NPV), diagnostic accuracy, error and kappa statistic) of conventional FNAC and ultrasound guided FNAC have been summarized on Table 2.

**Table 1:** conventional versus ultrasound guided FNAC and histology findings

Diagnostic method	Type of lesion	Histology		Total	p-value
		Malignant	Benign		
Conventional FNAC	Malignant	72	5	77	<0.00001
	Benign	11	112	123	
	Total	83	117	200	
Ultrasound guided FNAC	Malignant	51	0	51	<0.00001
	Benign	0	103	103	
	Total	51	103	154	

**Table 2:** diagnostic utility of conventional versus ultrasound guided FNAC in diagnosing breast malignancy

Diagnostic method	Sensitivity	Specificity	PPV	NPV	Accuracy	Error	Kappa statistic
Conventional FNAC	86.7	95.7	93.5	91.1	92.0	8.0	0.85
Ultrasound guided FNAC	100	100	100	100	100	0	1.0

## Discussion

Breast cancer is the most common malignant neoplasm and the second cause of death from cancer in women, with more than one million cases occurring worldwide annually. Breast lump, being benign or malignant is the main cause of anxiety to the patient and her family members [1,2]. Both ultrasound guided and conventional fine needle aspiration cytology (FNAC) have been used for diagnosing of breast malignancies among patients with palpable breast lumps. This study compared diagnostic utility of ultrasound guided versus conventional FNAC in pre-operative diagnosis of breast malignancy among patients with palpable breast masses. In this study, the proportion of breast cancer patients presenting with breast lumps was 37.9%, which is lower compared that was reported to be 43.4% in Tanzania, 45.7% in Pakistan and 67.0% in Uganda [4,7,17]. The lower proportion could be due to lack of awareness in the study community on primary prevention of breast cancer compared to developed world. The study conducted in Pakistan and Tanzania included only female patients with age above 35 years due to mammography inclusive criteria [4,17]. In the current study, a significant number of patients were below 25 years (24.9%), and this is a group with low risk of malignancy which could explain the different findings from previous study in Tanzania [17,19-22]. Furthermore, the study done in Uganda included only patients with suspicion of malignancy and who were above 18 years of age [7].

In the present study, patients with benign lesions were found to be younger than patients with breast cancer, this finding is in agreement with other studies [4,7,17]. The median age at diagnosis of patients with breast cancer in our study was 51 years, which is in keeping with other studies in developing countries [19,20], but lower than what is seen in Western countries where the median age at diagnosis is in the sixth decade of life [2,6,17]. In African women, the diagnosis of breast cancer is often made between 35 and 45 years of age which is approximately 10-15 years earlier than the peak incidence for Western countries [17]. The reasons for the early age of onset of breast cancer among black women are poorly understood but could probably be connected to the aggressive nature of the disease [17]. While numerous theories have been proposed to explain this age difference, including age at menarche, time at first pregnancy, parity, socio-demographic factors, body mass index and underlying genetic differences, none have been validated and more research is needed in this area [6]. It has been reported that the occurrence of breast cancer at young age is associated with poor prognosis and thus prognosis improves with age, the best prognosis is seen in patients over 75 years [7,17,19-22]. The fact that cancer in young age has different molecular features (hormonal receptors HER2; BRCA and p53 mutation) from those occurring in old age, and these factors are associated with poor prognosis [19-22]. In the present study, the left breast was mostly affected, and this finding is similar to other study done in North Sudan [13]. The reason for the left breast predilection to diseases is not well understood. From this study, the upper outer quadrant was more affected by disease.

Accurate diagnosis of a patient with a breast lump is of paramount importance and can help to avoid unnecessary surgical procedures and plan correct treatment options. Every patient with breast lump should undergo triple assessment consisting of clinical evaluation, mammography/ultrasound according to age, then fine needle aspiration cytology (FNAC) to make an early and accurate diagnosis. It has been shown that FNAC can reduce the number of open breast biopsies [4,7,17]. Fine needle aspiration cytology (FNAC) has been found to have sensitivity ranging from 82% to 97.5% and specificity of more than 99% [4,7]. In the present study, the diagnostic utility of conventional FNAC in terms of sensitivity, specificity, positive predictive value, negative predictive value and diagnostic accuracy were 86.7%, 95.7%, 93.5%, 91.1% and 92.0% respectively, showing almost perfect agreement with histological examination. These findings are comparable to the findings of other studies [8,15,17]. From this study, conventional FNAC false positive rate was lower than false negative rate (4.3% versus 13.3%). The higher rate of false negative could be attributed to non-cellular aspirates from very hard lumps, hemorrhagic aspirates from highly vascularized tumors and or aspirates from cystic lesions.

Another cause might be sampling error which may be reduced by localization of lesion using ultrasound guidance during aspiration [23]. Using ultrasound guided FNAC increases the diagnostic utility of FNAC and reduces the false positive and false negative rates of FNAC [23]. When performed adequately by a well-trained team, the diagnostic utility of ultrasound guided FNAC approaches 100% and definitive treatment can be safely started. A review paper found sensitivity values of ultrasound-guided FNAC between 25% and 95%; specificity values ranged from 97% to 100% false positives were between 1.4% and 1.6% false negatives were between 6% and 11% [24]. Aker *et al*. in a series of 733 cases of ultrasound-guided FNAC published the following results: sensitivity 98%, specificity 90%, overall accuracy 96%, and positive predictive value 96%, negative predictive value 94%, false positive 2.6%, and false negative 1.4% [25]. The latter findings are slightly different from the present study likely because of the differences in the sample sizes and nature of the study being a retrospective study in majority of patients. In the present study, the diagnostic utility of ultrasound guided FNAC in terms of sensitivity, specificity, positive predictive value, negative predictive value and diagnostic accuracy were all 100% respectively, with no false negative and false positive. The kappa statistic for ultrasound guided FNAC was 1.0 suggestive of almost perfect agreement with histological findings. Though this study was limited by inadequate documentation for the retrospective study leading to exclusion of some patients, it was able to demonstrate an excellent diagnostic utility of ultrasound guided FNAC for palpable breast lumps. It also highlighted the importance of multi-displinary team assessment of breast pathologies by a well trained and experienced team comprising of surgeons, radiologists and pathologists.

## Conclusion

Both ultrasound guided and conventional FNAC show almost perfect agreement with histology. However, ultrasound guided FNAC has a higher diagnostic utility relative to conventional FNAC in diagnosing breast malignancies.

### What is known about this topic

- Currently, image guided tissue sampling is the 'state of art' minimally invasive diagnostic method for suspected malignancies;
- Image guided tissue sampling provides accurate and direct visualization of lesions compared to the conventional (blind) method.

### What this study adds

- This research adds valuable knowledge on the role of ultrasound guided versus conventional (blind) fine needle aspiration cytology in diagnosing breast malignancies in a third world African tertiary hospital whereby, if breast malignancies are accurately detected early, will lead to significant decrease in the morbidity and mortality encountered in advanced disease;
- Image guided tissue sampling provides accurate and direct visualization of lesions with comparatively no false positives or false negative as shown in the study.
